# Risk of Second Primary Cancer among Prostate Cancer Patients in Korea: A Population-Based Cohort Study

**DOI:** 10.1371/journal.pone.0140693

**Published:** 2015-10-15

**Authors:** Jae Young Joung, Jiwon Lim, Chang-Mo Oh, Kyu-Won Jung, Hyunsoon Cho, Sung Han Kim, Ho Kyung Seo, Weon Seo Park, Jinsoo Chung, Kang Hyun Lee, Young-Joo Won

**Affiliations:** 1 Center for Prostate Cancer, National Cancer Center, Goyang, Korea; 2 Cancer Registration and Statistics Branch, National Cancer Center, Goyang, Korea; Taipei Medical University, TAIWAN

## Abstract

As patients with prostate cancer have a long life expectancy, there is increasing interest in predicting the risk of development of a second primary cancer (SPC), and we therefore designed this study to estimate the overall risk of developing SPCs among Korean prostate cancer patients. We used a population-based cohort from the Korean Central Cancer Registry composed of 55,378 men diagnosed with a first primary prostate cancer between 1993 and 2011. Standardized incidence ratios (SIRs) of SPCs were analyzed by age at diagnosis, latency period, period of diagnosis, and type of initial treatment. Survival analysis was stratified by development of SPC. Men with primary prostate cancer had an overall lower risk of developing an SPC [SIR = 0.75; 95% CI, 0.72−0.78], which was significant for SPCs of the esophagus, stomach, rectum, liver, gallbladder, bile duct, pancreas, larynx, lung, and bronchus. In contrast, there were significant increases in the risk of bladder and thyroid cancers, which tended to decrease after longer follow-up. Patients who received initial radiation therapy had an increased risk of subsequent rectal cancer, although this was still lower than that of the general male population. Other urinary tract cancers including those of the kidney, renal pelvis, and ureter tended to be associated with a higher risk of developing an SPC, but this difference did not reach statistical significance. The patients with prostate cancer and SPC had lower overall survival rates than those with one primary prostate cancer. Our findings suggest that men with prostate cancer have a 25% lower risk of developing an SPC in Korea, but a higher risk of developing subsequent bladder and thyroid cancers, which suggests the need for continued cancer surveillance among prostate cancer survivors.

## Introduction

In the United States, prostate cancer is currently the most common malignancy among men [1]. It is generally believed that the incidence of prostate cancer is lower in Asian countries [2], although in Korea its incidence is rapidly increasing compared to other cancers (12.3% annual increase) [3, 4]. Based on the recent report of the Korean Central Cancer Registry (KCCR), 8,952 new cases of prostate cancer were diagnosed in 2011 with an incidence of 27.4 per 100,000 men [3]. Moreover, incidental detection of prostate cancer is common in cystectomy specimens among patients with bladder cancer [5].

Due to the widespread adoption of the prostate-specific antigen (PSA) test, the majority of prostate cancers are diagnosed at an early stage. In Korea, this has led to an increase in survival of men diagnosed with prostate cancer. In fact, according to the current (2007−2011) estimate, five-year relative survival is now 92.0%, compared to 80.1% in prior years (2001−2005) [3]. As a consequence, although the life expectancy of prostate cancer patients has significantly increased, survivors are nonetheless at risk of developing a second primary cancer (SPC) after curative surgery and radiation therapy. Accordingly, there is increasing interest in predicting the risk of developing an SPC following the primary prostate cancer. Only a few studies have investigated the risk of developing an SPC [6–12]. In clinical practice, the identification of patients at increased risk of developing an SPC would help to optimize treatment of the primary prostate cancer and to promptly diagnose SPCs.

The objective of this study was to estimate the overall risk of developing SPCs among Korean patients with prostate cancer compared with the general population using a population-based cohort. The relative risk of developing an SPC was also analyzed according to the type of initial treatment along with the impact of developing an SPC on overall survival.

## Patients and Methods

### Study population and data source

The Korean Central Cancer Registry (KCCR) is a population-based cancer registry in Korea. The Ministry of Health and Welfare initiated a nationwide hospital-based cancer registry in 1980 [13]. Until 1998, the registry collected cancer cases annually from more than 180 hospitals in Korea and it is believed to include 80–90% of all cancer cases in Korea [13]. Since 1999, nationwide, population-based cancer incidence data has been generated systematically, and KCCR data from 1999 to 2002 have been published as “Cancer Incidence in Five Continents Vol. IX”, which reflects the completeness and validity of this data set [14]. Cancer diagnoses in the KCCR are coded using the International Classification of Disease for Oncology, 3^rd^ edition (ICD-O-3). Briefly, cancer registry information includes patient-related data (gender and age at diagnosis), tumor-related data (date of diagnosis; cancer site; morphology; and Surveillance, Epidemiology and End Results [SEER] summary stage), and primary treatment-related data (surgery, radiation therapy, hormonal therapy, and chemotherapy). The detailed contents of the KCCR have been published elsewhere [13].

The National Cancer Center institutional review board approved this study (NCC2015-0136).

In the present study, the cohort was composed of 55,378 men diagnosed with a first primary prostate cancer between 1993 and 2011. To avoid potential confusion, the following patients were excluded from the analysis: patients who presented with a SPC within two months of their first prostate cancer diagnosis (in order to exclude synchronous primary cancers), patients with subsequent prostate cancer after the diagnosis of another primary cancer, and patients for whom only death certificate information was available.

### Statistical analysis

Standardized incidence ratios (SIRs) and corresponding 95% confidence intervals (95% CIs) of SPCs following prostate cancer were analyzed to quantify the relative risk compared to men in the general population. The SIR as a measure of relative risk compares the incidence rate of the subsequent cancer of interest to the baseline incidence rate of that cancer in the general population. SIRs were calculated by dividing the observed number of second cancers by the expected number if the patients in the cohort demonstrated cancer rates equivalent to those for individuals in the general population. A Poisson distribution for the observed cases was used to calculate the 95% CIs. We calculated the person-years at risk from two months after the date of the initial prostate cancer diagnosis to the last known vital status, death, or the end of the study (December 31st, 2011), whichever occurred first. Analyses were stratified by age at prostate cancer diagnosis (early onset <65 years or late onset ≥65 years), latency period (<60 months, 60−119 months, or ≥120 months), and period of prostate cancer diagnosis (1993−2000 or 2001−2011). Age was dichotomized into <65 years and ≥65 years because this cutoff has commonly been used in demographic and epidemiologic studies, as well as statistics on the elderly (http://kosis.kr/). Results were also analyzed according to the type of initial treatment for prostate cancer. In this registry, the first treatment received within four months after diagnosis of prostate cancer was considered as the primary treatment.

Survival probabilities were calculated according to the presence of an SPC using the Kaplan-Meier method. Differences between groups were assessed using a log-rank test. All of the statistical tests were two-sided, and a P value <0.05 was considered statistically significant. We used SEER*Stat software (seer.cancer.gov/seerstat, version 8.1.5) to compute SIRs and the 95% CIs. Survival curves were generated and log-rank tests were performed using STATA version 11 (StataCorp LP, College Station, TX).

## Results

### Patient characteristics and risk of developing an SPC

The median age at diagnosis of prostate cancer was 70 years, and the majority of prostate cancers were diagnosed when the patient was 60−79 years old. Detailed characteristics of the cohort are presented in Table 1.

**Table 1:** Characteristics of patients with an initial cancer of the prostate diagnosed between 1993 and 2011.

**Categorical variables**	**N**	**%**
Men with prostate cancer	55,378	100.0
Period during which prostate cancer was diagnosed		
1993–1997	3,308	6.0
1998–2002	7,275	13.1
2003–2007	18,123	32.7
2008–2011	26,672	48.2
Number of men by age at 1^st^ primary prostate cancer diagnosis, years		
<40	96	0.2
40–49	629	1.2
50–59	5,390	9.7
60–69	20,278	36.6
70–79	22,227	40.1
≥80	6,758	12.2
Primary treatment for prostate cancer^a^		
Surgery	27,765	50.1
Radiation	3,407	6.2
Hormonal treatment	6,942	12.5
Chemotherapy	3,571	6.5
Number of men who developed a 2^nd^ primary cancer	2,578	4.7
Latency period between 1^st^ and 2^nd^ primary cancer		
<1 year	548	21.3
1–4 years	1,437	55.7
5–9 years	500	19.4
≥10 years	93	3.6
Number of men by age at 2^nd^ primary cancer diagnosis, years		
<40	3	0.1
40–49	9	0.3
50–59	87	3.4
60–69	669	26.0
70–79	1,339	51.9
≥80	471	18.3
**Continuous variables**	**Mean ormedianmedian**	**SD**
Mean age at diagnosis of 1st prostate cancer, years (SD)	69.8	8.6
Median age at diagnosis with 1st prostate cancer, years	70.0^b^	
Average follow up, months (SD)	42	37
Mean age at diagnosis of 2^nd^ primary cancer, years (SD)	73.1	7.5
Mean interval between 1^st^ and 2^nd^ cancers, years (SD)	3.3	2.9

^a^Treatment is defined as the treatment within 4 months after diagnosis of prostate cancer.

^b^Age at diagnosis with primary prostate cancer is expressed as median.

SD, standard deviation

Among 55,378 patients, 2,578 men (4.7%) developed a SPC during the observation period. Compared with the incidence of cancer in the general population, men with primary prostate cancer had an overall lower risk for developing an SPC [SIR = 0.75; 95% CI, 0.72−0.78)]. The reduction in risk was significant for cancers of the digestive system including those of the esophagus, stomach, rectum, liver, gallbladder, bile duct, and pancreas. There was also a reduced risk of cancers of the larynx, lung, and bronchus (Table 2).

**Table 2:** Risk of subsequent primary cancers by latency, age, and year of diagnosis following cancer of the prostate diagnosed between 1993 and 2011.

	Total		Latency (months)			Age (years)		Year of diagnosis	
			<60	60–119	≥120	< 65 years	≥ 65 years	1993–2000	2001–2011
	SIR (O/E)	95% CI	SIR (O/E)	SIR (O/E)	SIR (O/E)	SIR (O/E)	SIR (O/E)	SIR (O/E)	SIR (O/E)
**All subsequent cancers**	0.68# (2761/4067.46)	(0.65, 0.70)	0.68# (2115/3100.39)	0.67# (544/806.21)	0.63# (102/160.86)	0.78# (598/766.62)	0.66# (2163/3300.84)	0.60# (496/831.08)	0.70# (2265/3236.38)
**All subsequent cancers (excluding prostate)**	0.75# (2738/3649.18)	(0.72, 0.78)	0.75# (2094/2788.74)	0.75# (542/718.27)	0.72# (102/142.17)	0.84# (591/706.05)	0.73# (2147/2943.13)	0.64# (492/763.54)	0.78# (2246/2885.64)
**Buccal cavity, pharynx**	0.81 (54/66.39)	(0.61, 1.06)	0.81 (42/51.54)	0.56 (7/12.51)	2.14 (5/2.33)	0.79 (12/15.22)	0.82 (42/51.16)	0.85 (12/14.11)	0.80 (42/52.28)
**Digestive system**	0.72# (1523/2122.91)	(0.68, 0.75)	0.71# (1163/1629.53)	0.74# (306/412.93)	0.67# (54/80.46)	0.73# (320/441.06)	0.72# (1203/1681.85)	0.62# (273/437.28)	0.74# (1250/1685.63)
Esophagus	0.62# (65/104.95)	(0.48, 0.79)	0.69# (56/81.35)	0.45# (9/19.94)	0.00 (0/3.65)	0.58 (12/20.85)	0.63# (53/84.1)	0.46# (11/24.11)	0.67# (54/80.84)
Stomach	0.69# (549/797.53)	(0.63, 0.75)	0.67# (413/615.13)	0.78# (119/153.3)	0.58# (17/29.1)	0.79# (132/166.21)	0.66# (417/631.32)	0.64# (110/172.99)	0.70# (439/624.54)
Small intestine	0.91 (12/13.21)	(0.47, 1.59)	0.79 (8/10.08)	1.15 (3/2.61)	1.95 (1/0.51)	0.41 (1/2.46)	1.02 (11/10.75)	0.70 (2/2.84)	0.96 (10/10.37)
Colon	1.00 (315/315.71)	(0.89, 1.11)	1.05 (252/239.55)	0.81 (51/63.24)	0.93 (12/12.92)	0.96 (61/63.35)	1.01 (254/252.36)	0.74 (39/52.87)	1.05 (276/262.84)
Rectum, rectosigmoid	0.66# (164/249.18)	(0.56, 0.77)	0.65# (125/191)	0.66# (32/48.67)	0.74 (7/9.52)	0.70# (38/54.41)	0.65# (126/194.78)	0.57# (26/45.6)	0.68# (138/203.59)
Anus, anal canal	0.60 (3/5.01)	(0.12, 1.75)	0.27 (1/3.77)	1.95 (2/1.03)	0.00 (0/0.22)	1.34 (1/0.75)	0.47 (2/4.27)	0.77 (1/1.3)	0.54 (2/3.72)
Liver	0.56# (155/276.09)	(0.48, 0.66)	0.52# (113/216.46)	0.67# (34/50.47)	0.87 (8/9.16)	0.43# (32/74.67)	0.61# (123/201.42)	0.59# (35/59.61)	0.55# (120/216.48)
Gall bladder	0.60# (31/52.03)	(0.40, 0.85)	0.59# (23/39.05)	0.75 (8/10.71)	0.00 (0/2.27)	0.64 (5/7.83)	0.59# (26/44.2)	0.35# (4/11.5)	0.67# (27/40.53)
Bile ducts, other biliary	0.77# (141/184.13)	(0.64, 0.90)	0.74# (102/138.6)	0.88 (33/37.64)	0.76 (6/7.89)	0.79 (23/29.09)	0.76# (118/155.04)	0.77 (31/40.1)	0.76# (110/144.03)
Pancreas	0.72# (87/120.67)	(0.58, 0.89)	0.76# (69/91.12)	0.61 (15/24.47)	0.59 (3/5.07)	0.73 (15/20.56)	0.72# (72/100.1)	0.55# (14/25.28)	0.77# (73/95.39)
**Respiratory system**	0.64# (559/872.74)	(0.59, 0.70)	0.62# (410/662.58)	0.73# (128/175.13)	0.60# (21/35.03)	0.75# (105/139.35)	0.62# (454/733.4)	0.51# (99/192.69)	0.68# (460/680.06)
Nose, nasal cavity, ear	0.28 (2/7.05)	(0.03, 1.02)	0.37 (2/5.45)	0.00 (0/1.34)	0.00 (0/0.26)	0.68 (1/1.46)	0.18# (1/5.59)	0.00 (0/1.56)	0.36 (2/5.49)
Larynx	0.68# (35/51.17)	(0.48, 0.95)	0.55# (22/39.84)	1.35 (13/9.6)	0.00 (0/1.73)	0.55 (6/10.86)	0.72 (29/40.31)	0.65 (8/12.23)	0.69 (27/38.94)
Lung, bronchus	0.64# (521/810.9)	(0.59, 0.70)	0.63# (386/614.51)	0.70# (114/163.48)	0.64# (21/32.9)	0.77# (97/126.43)	0.62# (424/684.47)	0.51# (90/177.89)	0.68# (431/633.01)
**Male genital system**	0.08# (32/425.39)	(0.05, 0.11)	0.09# (27/317.05)	0.04# (4/89.37)	0.05# (1/18.98)	0.13# (8/61.73)	0.07# (24/363.67)	0.09# (6/69.3)	0.07# (26/356.1)
Prostate	0.05# (23/418.28)	(0.03, 0.08)	0.07# (21/311.65)	0.02# (2/87.94)	0.00# (0/18.69)	0.12# (7/60.57)	0.04# (16/357.71)	0.06# (4/67.54)	0.05# (19/350.74)
Testis	3.49 (2/0.57)	(0.42, 12.61)	4.45 (2/0.45)	0.00 (0/0.11)	0.00 (0/0.02)	6.52 (1/0.15)	2.39 (1/0.42)	0.00 (0/0.15)	4.76 (2/0.42)
**Male Breast**	0.37 (1/2.68)	(0.01, 2.08)	0.49 (1/2.02)	0.00 (0/0.54)	0.00 (0/0.12)	0.00 (0/0.44)	0.45 (1/2.24)	0.00 (0/0.66)	0.50 (1/2.02)
**Urinary system**	1.24# (304/246.13)	(1.10, 1.38)	1.31# (244/185.98)	1.04 (52/49.82)	0.77 (8/10.33)	1.69# (71/41.98)	1.14 (233/204.15)	1.16 (60/51.61)	1.25# (244/194.52)
Urinary bladder	1.26# (197/156.56)	(1.09, 1.45)	1.32# (155/117.32)	1.11 (36/32.35)	0.87 (6/6.88)	2.03# (45/22.17)	1.13 (152/134.39)	1.39# (50/35.92)	1.22# (147/120.63)
Kidney parenchyma	1.15 (74/64.63)	(0.90, 1.44)	1.26 (63/49.82)	0.73 (9/12.39)	0.83 (2/2.41)	1.22 (19/15.53)	1.12 (55/49.09)	0.46 (5/10.96)	1.29# (69/53.66)
Renal pelvis, other urinary	1.32 (33/24.95)	(0.91, 1.86)	1.38 (26/18.83)	1.38 (7/5.07)	0.00 (0/1.04)	1.64 (7/4.28)	1.26 (26/20.67)	1.06 (5/4.73)	1.38 (28/20.22)
Ureter	1.30 (16/12.34)	(0.74, 2.11)	1.41 (13/9.24)	1.17 (3/2.56)	0.00 (0/0.54)	2.01 (4/1.99)	1.16 (12/10.35)	0.44 (1/2.26)	1.49 (15/10.09)
**Soft tissue including heart**	1.08 (13/12)	(0.58, 1.85)	0.99 (9/9.07)	1.24 (3/2.41)	1.92 (1/0.52)	1.29 (3/2.32)	1.03 (10/9.68)	0.42 (1/2.4)	1.25 (12/9.61)
**Melanoma of the skin**	1.14 (9/7.87)	(0.52, 2.17)	1.18 (7/5.95)	1.26 (2/1.59)	0.00 (0/0.33)	0.00 (0/1.44)	1.40 (9/6.44)	0.68 (1/1.47)	1.25 (8/6.4)
**Brain, CNS**	0.77 (14/18.21)	(0.42, 1.29)	0.72 (10/13.95)	0.85 (3/3.55)	1.41 (1/0.71)	2.04 (8/3.91)	0.42# (6/14.3)	0.84 (3/3.59)	0.75 (11/14.62)
**Thyroid**	2.06# (80/38.87)	(1.63, 2.56)	2.22# (69/31.05)	1.34 (9/6.71)	1.80 (2/1.11)	2.38# (37/15.53)	1.84# (43/23.34)	2.10 (9/4.28)	2.05# (71/34.59)
**Lymphatic, hematopoietic**	0.85 (103/121.2)	(0.69, 1.03)	0.86 (80/92.52)	0.84 (20/23.91)	0.63 (3/4.77)	0.99 (24/24.36)	0.82 (79/96.83)	0.90 (21/23.24)	0.84 (82/97.96)

^#^ *P* <0.05

SIR, standardized incidence ratio; O, observed number of second primary cancers; E, expected number of second primary cancers; O/E, ratio of observed number of second primary cancers to the number of expected cancers; 95% CI, 95% confidence interval; CNS, central nervous system

Conversely, there was a significant increase in the risk of bladder and thyroid cancer. Other cancers of the urinary tract, including those of the kidney, renal pelvis, and ureter, tended to be associated with a higher risk of SPC, although this difference did not reach statistical significance.

### Latency

Following a diagnosis of prostate cancer, the risk of bladder cancer as an SPC tended to decrease over time (Table 2). For patients examined within 60 months after diagnosis of prostate cancer, there was an increased risk of bladder cancer [SIR = 1.32]. For patients followed for 60−119 months, the risk decreased to an SIR of 1.11. Finally, the SIR decreased to 0.87 in patients after an even longer follow-up period (≥120 months). Similar findings were seen for thyroid cancer.

### Age at diagnosis of prostate cancer

To identify the impact of age at diagnosis of prostate cancer on the risk of developing an SPC, patients were stratified by age into an early-onset group (<65 years) and a late-onset group (≥65 years). Men in the early-onset group had a relatively higher risk of both bladder (SIR = 2.03) and thyroid cancer (SIR = 2.38) compared with men in the late-onset group (SIR = 1.13 for bladder cancer and 1.84 for thyroid cancer). For tumors of the CNS, the early-onset group showed a higher risk of subsequent CNS tumors, although this difference was not statistically significant. In contrast, the late-onset group showed a significantly lower risk of developing subsequent CNS tumors (SIR = 0.42).

### Period of diagnosis of prostate cancer

The data collected in the KCCR cover around 20 years, during which the techniques used for diagnosis and treatment have changed. Thus, two different periods were considered (1993−2000 vs. 2001−2011). However, the only significant difference observed between these periods was an increased risk of kidney cancer in patients with prostate cancer in 2001−2011 (Table 2). This difference may be related to an increased public participation in cancer screening as a result of private programs compared to 1993−2000.

### Initial therapy for prostate cancer

To investigate the impact of initial therapy for prostate cancer, differences in the risk of developing an SPC according to initial treatment including surgery, radiation therapy, hormonal therapy, and chemotherapy were explored (Table 3).

**Table 3:** Risk of subsequent primary cancers by primary treatment^a^ for prostate cancers diagnosed between 1993 and 2011.

	Total		Surgery	Radiation	Hormone	Chemotherapy
	SIR (O/E)	95% CI	SIR (O/E)	SIR (O/E)	SIR (O/E)	SIR (O/E)
**All subsequent cancers**	0.68# (2761/4067.46)	(0.65, 0.70)	0.71# (1411/1999.59)	0.71# (189/267.38)	0.78# (361/464.64)	0.76# (183/240.95)
**All subsequent cancers (excluding prostate)**	0.75# (2738/3649.18)	(0.72, 0.78)	0.78# (1401/1800.95)	0.79# (189/240.59)	0.87# (358/413.45)	0.83# (180/216.13)
**Buccal cavity, pharynx**	0.81 (54/66.39)	(0.61, 1.06)	1.01 (34/33.8)	1.12 (5/4.46)	0.82 (6/7.31)	1.02 (4/3.9)
**Digestive system**	0.72# (1523/2122.91)	(0.68, 0.75)	0.72# (758/1058.41)	0.77# (108/140.51)	0.82# (197/239.13)	0.77# (96/125.25)
Esophagus	0.62# (65/104.95)	(0.48, 0.79)	0.51# (27/52.8)	0.14# (1/7.07)	0.78 (9/11.47)	0.32 (2/6.21)
Stomach	0.69# (549/797.53)	(0.63, 0.75)	0.72# (288/400.17)	0.72# (38/53.05)	0.71# (63/88.44)	0.70# (33/47.03)
Small intestine	0.91 (12/13.21)	(0.47, 1.59)	0.62 (4/6.47)	1.15 (1/0.87)	1.34 (2/1.49)	1.28 (1/0.78)
Colon	1.00 (315/315.71)	(0.89, 1.11)	0.95 (147/155.26)	1.11 (23/20.65)	1.31 (49/37.31)	1.18 (22/18.59)
Rectum, rectosigmoid	0.66# (164/249.18)	(0.56, 0.77)	0.67# (83/124.69)	1.03 (17/16.43)	0.74 (21/28.55)	0.82 (12/14.63)
Anus, anal canal	0.60 (3/5.01)	(0.12, 1.75)	0.41 (1/2.42)	0.00 (0/0.31)	1.81 (1/0.55)	0.00 (0/0.3)
Liver	0.56# (155/276.09)	(0.48, 0.66)	0.54# (77/142.91)	0.64 (12/18.76)	0.67 (20/29.82)	0.74 (12/16.2)
Gallbladder	0.60# (31/52.03)	(0.40, 0.85)	0.69 (17/24.73)	0.30 (1/3.36)	0.50 (3/6.01)	0.64 (2/3.12)
Bile ducts, other biliary	0.77# (141/184.13)	(0.64, 0.90)	0.85 (75/88.35)	0.84 (10/11.89)	0.57# (12/21.15)	0.73 (8/10.98)
Pancreas	0.72# (87/120.67)	(0.58, 0.89)	0.65# (38/58.37)	0.64 (5/7.82)	1.23 (17/13.87)	0.56 (4/7.17)
**Respiratory system**	0.64# (559/872.74)	(0.59, 0.70)	0.67# (284/422)	0.66# (38/57.52)	0.71# (71/99.55)	0.73# (38/52.2)
Nose, nasal cavity, ear	0.28 (2/7.05)	(0.03, 1.02)	0.28 (1/3.54)	2.10 (1/0.48)	0.00 (0/0.79)	0.00 (0/0.42)
Larynx	0.68# (35/51.17)	(0.48, 0.95)	0.81 (21/25.93)	0.29 (1/3.46)	0.36 (2/5.51)	0.99 (3/3.03)
Lung, bronchus	0.64# (521/810.9)	(0.59, 0.70)	0.67# (262/390.76)	0.67# (36/53.35)	0.74# (69/92.87)	0.72 (35/48.53)
**Male genital system**	0.08# (32/425.39)	(0.05, 0.11)	0.06# (12/202.12)	0.00# (0/27.24)	0.06# (3/51.97)	0.20# (5/25.25)
Prostate	0.05# (23/418.28)	(0.03, 0.08)	0.05# (10/198.64)	0.00# (0/26.79)	0.06# (3/51.19)	0.12# (3/24.82)
Testis	3.49 (2/0.57)	(0.42, 12.61)	3.37 (1/0.3)	0.00 (0/0.04)	0.00 (0/0.06)	29.00 (1/0.03)
**Male Breast**	0.37 (1/2.68)	(0.01, 2.08)	0.77 (1/1.31)	5.92 (1/0.17)	0.00 (0/0.3)	0.00 (0/0.16)
**Urinary system**	1.24# (304/246.13)	(1.10, 1.38)	1.41# (168/118.86)	1.25 (20/15.99)	1.69# (48/28.35)	1.5 (22/14.65)
Urinary bladder	1.26# (197/156.56)	(1.09, 1.45)	1.55# (115/73.99)	1.29 (13/10.06)	1.56# (28/17.96)	2.02# (19/9.4)
Kidney parenchyma	1.15 (74/64.63)	(0.90, 1.44)	1.13 (37/32.81)	1.63 (7/4.29)	1.75 (13/7.43)	0.53 (2/3.77)
Renal pelvis, other urinary	1.32 (33/24.95)	(0.91, 1.86)	1.33 (16/12.06)	0.00 (0/1.63)	2.37 (7/2.95)	0.67 (1/1.49)
Ureter	1.30 (16/12.34)	(0.74, 2.11)	1.53 (9/5.88)	0.00 (0/0.8)	1.35 (2/1.48)	0.00 (0/0.74)
**Soft tissue including heart**	1.08 (13/12)	(0.58, 1.85)	1.03 (6/5.84)	1.28 (1/0.78)	2.16 (3/1.39)	1.41 (1/0.71)
**Melanoma of the skin**	1.14 (9/7.87)	(0.52, 2.17)	1.32 (5/3.79)	0.00 (0/0.52)	3.24 (3/0.92)	0.00 (0/0.47)
**Brain, CNS**	0.77 (14/18.21)	(0.42, 1.29)	0.67 (6/9.01)	2.47 (3/1.21)	1.44 (3/2.08)	0.92 (1/1.08)
**Thyroid**	2.06# (80/38.87)	(1.63, 2.56)	2.52# (55/21.86)	1.98 (5/2.52)	1.17 (5/4.28)	0.94 (2/2.13)
**Lymphatic, hematopoietic**	0.85 (103/121.2)	(0.69, 1.03)	0.93 (56/59.95)	0.25# (2/8)	1.00 (14/13.97)	0.98 (7/7.17)

^#^ *P* <0.05

^a^Treatment was considered to have been for the original prostate cancer if it was given within 4 months of its diagnosis.

SIR, standardized incidence ratio; O, observed number of second primary cancers; E, expected number of second primary cancers; O/E, ratio of observed number of second primary cancers to the number of expected cancers; 95% CI, 95% confidence interval; CNS, central nervous system

The risk of SPC was significantly lower than that of the general population with each type of treatment (Table 3). Conversely, the risk of bladder cancer was consistently higher than that of the general population for all therapeutic options. However, there was no significant association between bladder cancer risk and use of radiation therapy. The risk of thyroid cancer increased only in the group receiving surgery. The overall risk of developing subsequent rectal cancer was lower (SIR = 0.66). In the 3,407 (6.2%) patients who received radiation therapy as initial therapy, there was a modestly higher risk of subsequent rectal cancer (SIR = 1.03) compared to patients undergoing other types of treatment, but this was not statistically significant.

### Survival analysis

**Fig 1:**
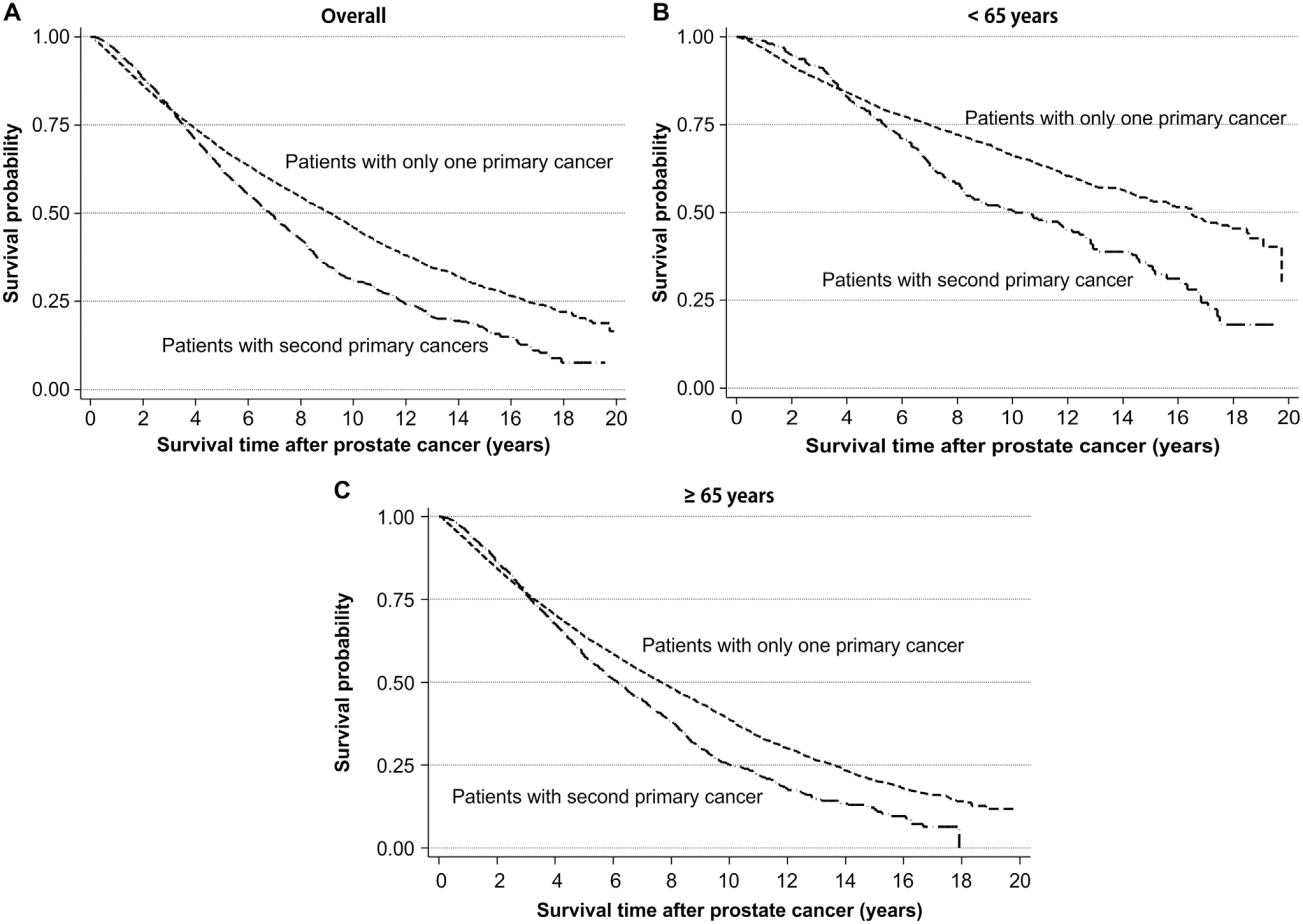
Kaplan-Meier curves showing survival after diagnosis of prostate cancer according to the presence of a second primary cancer. (A) All patients, (B) patients aged <65 years, (C) patients aged ≥65 years.

**Fig 2:**
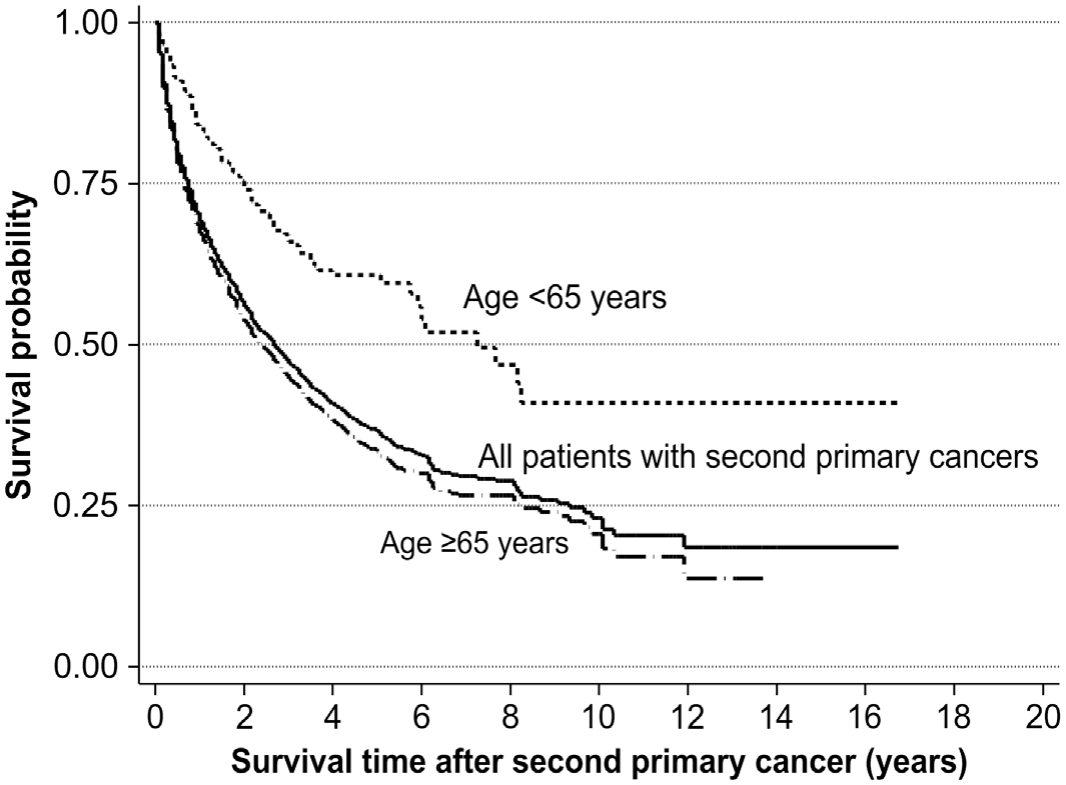
Kaplan-Meier curves showing survival after a second primary cancer.

During the 10-year follow-up period, 1,378 of the 2,578 prostate cancer patients died (data not shown). The 10-year overall survival (OS) rate in patients with SPC was 30.8%, which was lower than the 45.8% 10-year OS rate in those without SPC (log-rank test, *p* < 0.001; Fig 1A). This pattern of survival was similar in the both age groups (Fig 1B and 1C). The 10-year OS rate was 23.1% following diagnosis of an SPC. Patients in the early-onset group had a 10-year OS rate of 41.0%, which was considerably higher than the 20.6% 10-year OS rate observed for late-onset patients (log-rank test, *p* < 0.001; Fig 2).

## Discussion

In this cohort of prostate cancer patients, we found that there was a reduced overall risk of developing an SPC, which is consistent with previous reports [15–20]. While the mechanisms responsible for this reduced risk are unclear, they may be related in part to the age of patients at the time of diagnosis of prostate cancer.

In contrast, the increased risk of cancers of the urinary tract and thyroid may be explained by the increased use of cancer screening tests, which have a substantial impact on the early detection of malignancy [18]. In the present study, the incidence of SPC was higher during the 60 months following prostate cancer diagnosis than during later periods, especially for bladder and thyroid cancers. A possible relationship between increased screening and the growing number of cancer diagnoses is further supported by our finding that the risk of subsequent kidney cancer was higher during the more recent period (2001–2011), when radiologic examinations for cancer detection were commonly performed in Korea.

In this study, the overall risk of rectal cancer was lower in patients with prostate cancer compared to the general population, with the exception of patients that initially underwent radiation therapy, who tended to have an increased risk of subsequent rectal cancer. In general, exposure to ionizing radiation is considered to be a potential cause of cancer. Regarding the risk of SPC with respect to the treatment method used for prostate cancer, some studies have shown that external beam radiation therapy for prostate cancer does not increase the risk of SPC, including bladder cancer [21, 22].

From the SEER database, there are a few large studies that include patients diagnosed in the pre-PSA era. Nonetheless, similar results were reported, most notably that men receiving radiotherapy for localized prostate cancer had a 34−100% increase in the risk of bladder cancer compared to patients undergoing radical prostatectomy [23–26]. It was also observed that men who received external beam radiation therapy had a significantly increased risk of rectal cancer [23, 24, 26]. Based on more recent data from the SEER database, Davis et al. evaluated 441,504 men diagnosed with prostate cancer between 1992 and 2010 and found that prostate cancer survivors had a lower risk of SPC compared to the general population (SIR = 0.60). The incidence of many cancers including leukemia, and those of the oral cavity, pharynx, esophagus, stomach, colon, rectum, liver, gallbladder, and lung were also significantly lower. However, these patients had a greater risk of bladder, kidney, endocrine, and soft tissue cancers [27]. Through analysis of the Cancer of the Prostate Strategic Urologic Research Endeavor (CaPSURE) database from 1989 to 2003, Boorjian et al. found that 1.5% of bladder cancer patients had previously had prostate cancer, and also demonstrated that radiation therapy increased the risk of bladder cancer [28]. Our findings were similar to those based on studies of the SEER database, in which a lower risk of SPCs of the esophagus, stomach, rectum, liver, gallbladder, and lung were found. In addition, there was an increased risk of bladder and kidney cancer. However, the small number of patients who received radiation therapy could have obscured a significant association between radiation therapy and the risk of bladder cancer.

A recent systematic review reported that a slightly increased risk of radiation-induced SPC was present in several studies, particularly those with longer follow-up. In terms of the different techniques used for radiation therapy, it was not possible to draw any firm conclusions about the impact of intensity modulating radiation therapy and brachytherapy due to insufficient clinical data and the marked heterogeneity of published data [29].

The present study has some limitations. The KCCR does not record environmental factors, lifestyle factors, comorbidities other than cancers, PSA levels at diagnosis, or Gleason scores. Although the KCCR has collected data on SEER summary stages (localized, regional, and distant) since 2005, this information was not available for our analysis of SPCs, both because of the period of our study and the high rates of missing stage information in 2005–2011. Moreover, because of the small number of patients who received radiation therapy in this cohort, the influence of radiation therapy on subsequent cancer risk could not be investigated fully. Instead, we focused on the common etiologies of carcinogenesis for prostate cancer and SPCs of urinary tract. The present study was also limited by the short follow-up period. The median duration of follow-up was 42 months in the entire study cohort. This duration is relatively short in comparison with the follow-up periods of previous studies. Further studies with longer follow-up periods will be needed to estimate the precise risk of developing SPC and to overcome surveillance bias. Although the present study has limitations, it is the first nationwide analysis to estimate the risk of SPC among prostate cancer patients in Korea and provide basic information on the characteristics and survival rates of SPC patients with primary prostate cancer. Moreover, the present study is based on a population-based registry that covers more than 97% of all recent cancer cases. This allows the results to be generalized to all Korean men. The current findings also suggest that patients who were exposed to radiation for cancer treatment are at a higher risk of developing rectal cancer. Despite inconsistent results in previous studies, this suggests that additional surveillance for early detection of rectal cancer is needed in patients who received radiation therapy for prostate cancer [23, 30–32].

## Conclusions

The risk of developing an SPC after prostate cancer is lower than in the general population. However, patients with prostate cancer remain at increased risk of some malignancies, particularly bladder and thyroid cancer. The overall risk of rectal cancer is lower compared with the general population, although patients who received radiation therapy had a greater risk of subsequent rectal cancer than the general population. In survival analysis, the presence of an SPC had a negative impact on the OS of prostate cancer patients independently of the patient’s age. From a clinical perspective, these results suggest the need for continued cancer surveillance among prostate cancer survivors.
